# New Method of Auscultatory Percussion of the Chest

**Published:** 1917-08

**Authors:** 


### ABSTRACTS.

**New Method of Auscultatory Percussion of the Chest.** Robert M. Alexander, N. Y. Med. Jour., June 30. Instead of employing the usual method of determining continuity of any area, of the same anatomic and pathologic nature, by noting the different intensity when the stethoscope and percussing finger (or as in the editor's modification, a tuning fork, electric buzzer, etc.) or both over the same area or not, the author places the stethoscope over the upper part of the gladiolus of the sternum, or over the upper spinous processes and finds that a consolidated area transmits a higher pitched percussion sound to the stethoscope. He also combines the location of stethoscope and percussor by using a double diaphragm stethoscope and percussing over the outer diaphragm. This later method, we would suppose could be used in locating organs generally.

